# Identifying cognitive impairment in older adults using machine learning on combined fNIRS and motion data during an upper extremity dual task function

**DOI:** 10.21203/rs.3.rs-9519467/v1

**Published:** 2026-07-15

**Authors:** Kelsi Petrillo, Nima Toosizadeh

**Affiliations:** a Department of Rehabilitation and Movement Sciences, School of Health Professions, Rutgers University, 65 Bergen St, Newark, NJ 07107; b Department of Neurology, Rutgers Health, Rutgers University, 210 Somerset St, New Brunswick, NJ 08901; c Brain Health Institute, Rutgers University, 683 Hoes Lane West, Piscataway, NJ 08854

**Keywords:** machine learning, dual task, fNIRS, motor variability, resting state, dementia

## Abstract

It is critical that dementia clinical interventions begin early in the disease progression to be effective. Similar clinical manifestations may be observed in both cognitively healthy older adults and older adults with early-stage cognitive impairment, posing a challenge for early disease identification. This study explored classification models using a combination of motor-(gyroscope) and brain- (functional near infrared spectroscopy (fNIRS)) based features for potential use in screening cognitive impairment in older adults. Cognitively normal older adults (CNOA, n = 43; age = 75.47 ± 7.15) and cognitively impaired older adults (CIOA, n = 32; age = 75.90 ± 7.48) completed a 3-minute resting period followed by 3-minute upper extremity dual task function (UEF) involving simultaneous serial subtraction and elbow flexion. The selected features included motor variability and fNIRS anterior prefrontal cortex connectivity outcomes. Logistic regression, support vector machine (SVM), and bootstrap aggregated decision trees predicted the cognitive classification of participants. Cross-validation results suggest SVM models had superior performance with an average accuracy of 76%, Receiver Operating Characteristic - Area Under Curve (ROC-AUC) of 0.86, and F1 score of 69. When used with classification algorithms, the UEF dual task may offer an objective technique for early dementia identification.

## 1. Introduction

Dementia is a general term to describe diseases leading to symptoms that disrupt daily life such as memory loss, mood changes, problem solving, language, and more [1]. Alzheimer’s disease (AD) is the most common form of dementia, followed by Lewy body dementia (LBD) and vascular dementia [2, 3]. Recently, disease modifying therapeutics, such as lecanemab, have been approved for use in treating AD [4, 5]. However, it is critical that interventions begin early in the disease progression to be effective. Lecanemab, for instance, is recommended for use in those with mild cognitive impairment (MCI) or early-stage AD, but has not been shown to have efficacy in those with advanced AD [4]. Similarly, there are no approved disease-modifying treatments for LBD, but the disease leads to rapid degeneration and early intervention is critical for effective therapeutics [6].

Age is the most influential risk factor for developing a form of dementia and it is estimated that 80% of dementia cases are individuals over the age of 75 [2]. Additionally, cognitive decline and neurodegeneration increase with aging, even in those who never develop dementia [7]. Therefore, similar clinical manifestations, such as memory loss, may be observed in both healthy older adults and older adults with early-stage cognitive impairment. Shared characteristics between these two pose a challenge for identifying diseases early in their progression. There are many neuropsychological tests, often used to measure performance of cognitive domains, available to screen for cognitive impairment. Popular tests include the Mini-Mental State Examination (MMSE), clock drawing test, Mini-cog verbal fluency, and the Montreal Cognitive Assessment (MoCA). After screening, additional follow-up testing is necessary to confirm dementia and diagnose specific forms. Additional tests can include blood work, positron emission tomography (PET) imaging, and cerebrospinal fluid (CSF) samples [8]. With all the progress in screening dementia, unfortunately, it is estimated that more than half of dementia cases are undiagnosed [9]. It is critical to establish an objective and cost-effective cognitive impairment screening mechanism to help improve early identification.

Functional neuroimaging techniques have become an increasingly popular method of investigating changes in brain function due to cognitive impairment. In contrast to traditional structural imaging techniques, functional measures have been suggested to detect early stages of dementia before significant neurodegeneration occurs [10]. These techniques are also generally noninvasive and safe, making them clinically viable. Functional magnetic resonance imaging (fMRI) and electroencephalography (EEG) are two common examples of such techniques that have been previously used to identify differences between healthy older adults and those with age-related dementias [10, 11]. Functional near-infrared spectroscopy (fNIRS) is a relatively novel neuroimaging technique that records changes in hemodynamic activity over cortical brain regions. Compared to EEG, fNIRS has improved spatial resolution and does not require gel solutions [12]. Compared to fMRI, fNIRS has advantages in portability for use in task-based recordings, improved temporal resolution, and is relatively low cost [13].

We previously developed a novel upper-extremity function (UEF) dual task, involving elbow flexion and serial subtraction by threes, which has shown potential for screening cognitive impairment in older adults [14]. Counting is selected as the cognitive component as it involves working memory and executive functioning [15]. Although gait is commonly used as the motor component of dual tasks, using UEF may be preferrable for both patients and clinics, as it has no fall risk, can be executed by those with limited mobility, and requires minimal space [16]. Measures of motor variability during the UEF dual task demonstrated improved performance on detecting cognitive impairment compared to a gait dual task [17]. Additionally, motor performance measures from the UEF dual task were significantly associated with neuropsychological test scores, including MMSE, mini-cog verbal fluency, and MoCA [18].

Finally, differences in brain function outcomes during the UEF dual task between cognitively healthy and cognitively impaired older have been identified [19, 20].

fNIRS data has become increasingly popular in machine learning classification models [21]. Classification models can be especially useful for screening or identifying various disorders. For instance, recent work has used classification models based on fNIRS predictors to distinguish between those with Parkinson’s disease and age-matched healthy controls [22], and to explore early autism spectrum diagnosis [23]. Within our own previous work, statistical models have been explored and validated to differentiate between cognitively healthy and cognitively impaired older adults using wearable sensor-based motor outcomes from the UEF dual task as features [14, 17, 24]. The goal of the current study was to investigate classification models using a combination of motor- and brain- based features measured by wearable sensors during a resting state and the UEF dual task for potential use in screening cognitive impairment in older adults.

## 2. Methods

### 2.1. Participants

This study was approved by the Rutgers University institutional review board. All participants gave informed consent for study participation and the publication of images in an online open-access publication. Research was performed in accordance with the Declaration of Helsinki. Data from age-matched cognitively normal older adults (CNOA) and cognitively impaired older adults (CIOA) was collected from two locations. Participants were recruited from Tucson, Arizona and its surrounding areas during January 2022 to June 2023. Additionally, participants were recruited from Newark, New Jersey and its surrounding areas from February 2024 to September 2025. Inclusion criteria for participants were: 1) age of 65 or greater; 2) ability to understand study instructions; and 3) English language proficiency. Exclusion criteria were: 1) diagnosed diseases associated with severe motor performance deficits including stroke, Parkinson’s disease, tumors, traumatic brain injury, multiple sclerosis, and seizure disorder; 2) severe speech disorders; 3) severe upper-extremity disorders (e.g., elbow bilateral fractures or rheumatoid arthritis); and 4) severe or unstable medical disorders or conditions, including schizophrenia, bipolar disorder, psychotic features of depression, or any condition that makes the patient unsuitable for participating in the study.

Primary care physicians provided the cognitive status of participants and was confirmed using MoCA scores. Participants with a score of 24 or above were considered CNOA, and those with any score below 24 were grouped as CIOA. Although MoCA guidelines suggest a cutoff score of 26, recent research found superior classification and specificity for suspected neurocognitive disorders using a score of 24 [25].

### 2.2. Clinical and Neuropsychological Measures

The MoCA assessed cognitive domains including memory, language, executive functions, visuospatial skills, calculation, abstraction, attention, concentration, and orientation [26]. The Charlson Comorbidity Index (CCI) assessed comorbidity, as it is associated with an increased risk of cognitive impairment [27, 28]. The Patient Health Questionnaire-9 (PHQ-9) scaled depression, which is associated with cognitive deficits in executive function, memory, and attention [29, 30].

### 2.3. Data Recording

**Figure 1.**
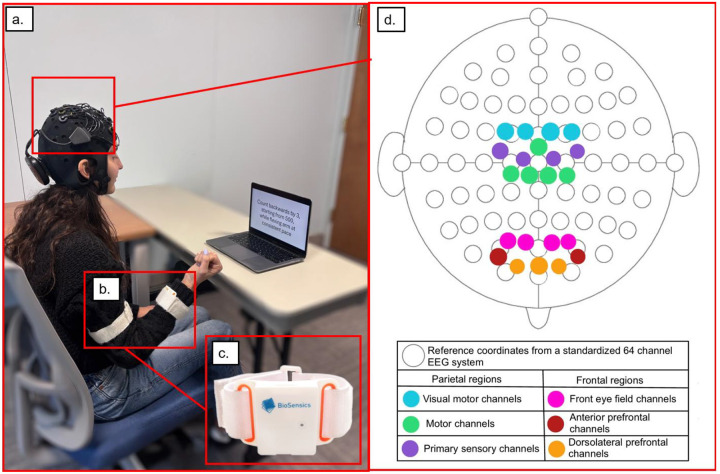
Experimental paradigm and devices. a. Dual task paradigm. b. Motion sensor placement. c. Wearable gyroscope motion sensor. d. Location of fNIRS channels in reference to the International Federation of Clinical Neurophysiology's 10–20 system electrode locations (white) [31]. Channel groupings based on the corresponding Broadmann’s areas are indicated by cyan, green, purple, pink, maroon, and orange for visual motor, motor, primary sensory, front eye fields (FEF), anterior prefrontal (aPFC), and dorsolateral prefrontal (dlPFC), respectively.

Participants completed a 3-minute resting period followed by a 3-minute UEF dual task while equipped with an fNIRS device and two wearable motion sensors attached to the right wrist and bicep (Figure 1). Participants sat with their eyes fixed on a computer screen displaying task instructions for the duration of the measurement (Figure 1). For the 3-minute rest period, participants were asked to remain still and clear their minds. During the 3-minute UEF dual task, participants were instructed to perform verbal serial 3 subtraction starting from a 3-digit number while consistently flexing their right arm at a self-selected pace. There was no break between the rest and task periods. During the dual task, audio recordings were taken and later used to generate counting sequences.

### 2.4. Devices

Brain activity was recorded using an Artinis Brite MK24 fNIRS device equipped with 10 emitters (763 nm and 842 nm) and 8 detectors. An optode template obtained from Artinis (Brite24 1×9+1×13) was used to set up the cap, such that there were 22 total channels (Figure 1). The distance between all transmitter and receiver pairs was 3 centimeters. Channels were grouped by brain regions, as defined by the Broadmann’s areas most associated with the scalp area under each channel. These associations were determined by matching channel locations to the standardized International Federation of Clinical Neurophysiology's 10–20 EEG system [9]. The identified Broadmann’s areas included the primary sensory, visual motor, motor, front eye fields (FEF), anterior prefrontal cortex (aPFC), and the dorsolateral prefrontal cortex (dlPFC) (Figure 1). The sampling rate of the device was set to 50 Hz. Upper-arm and forearm angular velocity were measured using two wearable motion sensors (Figure 1) with a sampling rate of 100 Hz (tri-axial wearable gyroscope sensor, Bio-Sensics LLC, Cambridge, MA).

### 2.5. Motion Data Processing

Only the first 60 seconds of motor data was analyzed to minimize confounding fatigue effects. Gyroscope data underwent initial noise and drift removal through a first-order high-pass Butterworth filter with a cutoff of 2.5 Hz. Subsequently, integration and differentiation with respect to time were performed to compute joint angles and angular acceleration, respectively. Three different peak dependent measures were explored, including 1) flexibility variability, defined as the coefficient of variation (COV) of the flexion angle range of motion; 2) flexion variability, defined as COV of time distances between consecutive angular velocity peaks; and 3) flexion number, defined as the total number of flexions [32]. Additionally, to explore nonlinear signal features, sample entropy was calculated to assess motor variability:

$$\text{SamEn}(m,r,N)=-\text{ln}\frac{P_{m+1}(r)}{P_{m}(r)}$$

defined as the probability that a sequence of “M” that repeats itself within a tolerance “r” for a certain time-window will also repeat itself for “*M*+1” points [32].

### 2.6. fNIRS Data Processing

Raw light intensity was converted to oxygenated hemoglobin concentration (HbO) using Oxysoft software (Artinis Medical Systems, Gelderland, Netherlands). The differential path-length factor was set to 6.61 for all participants. Signals were segmented using Oxysoft to separate the rest and task into two time series. Then, using the Oxysoft2Matlab tool (v1.83) data were exported to file type NIRS-SPM [33]. Data were imported into the NIRS-KIT toolbox for data preparation [34]. Temporal derivative distribution repair (TDDR) motion correction was applied to trials containing motion artifacts [35]. Additionally, saturated signals were excluded. Two of the main sources of physiological noise in fNIRS signals are heartbeat (1.0–1.5 Hz) and respiration (0.2–0.5 Hz) [36]. To remove noise, data were filtered using two different methods. To filter the data, two distinct preprocessing streams were used. For correlation-based outcomes, data was prepared using a 0.20 Hz 1^st^ order low pass filter to remove physiological artifacts but preserve slow hemodynamic fluctuations that contribute to time-domain variance for use in correlation-based outcomes [37]. For coherence-based outcomes, data was prepared using a 0.01–0.20 Hz 3^rd^ order infinite impulse response (IIR) bandpass filter to remove both physiological artifacts and low-frequency drift for use in coherence-based outcomes [38].

Using the fNIRS time series that were filtered with a low pass filter, the Pearson’s correlation coefficient (r) between every possible channel pair was calculated using MATLAB, such that a 22×22 matrix was obtained [39]. The Pearson’s correlation coefficient is a measure of linear dependency between two channels, and values can range from −1 to 1. Also, for each condition, the magnitude-squared coherence between all bandpass filtered channel pairs was calculated and averaged across all frequencies, such that a 22×22 matrix was obtained. Magnitude squared coherence is a measure of how well channels correspond at each frequency, and values can range between 0 and 1. The magnitude-squared coherence is expressed as: [40]

$$C_{xy}(f)=\frac{{\left|P_{xy}(f)\right|}^{2}}{P_{xy}(f)P_{yy}(f)}$$

Where *x* and *y* are two times series, *P*_*xx*_(*f*) and *P*_*yy*_(*f*) are the power spectral densities of *x* and *y*, respectively, and *P*_*xy*_(*f*) is the cross power spectral density between *x* and *y*. Magnitude-squared coherence may be referred to as coherence throughout the remainder of the paper.

To generate each subject’s graph, correlation and coherence value matrices (cutoff = 0.50) were used to generate undirected adjacency matrices [41]. To calculate graph-based outcomes, the Brain Connectivity Toolbox was used in MATLAB [42]. Degree was calculated by counting the functional connections for each channel [43]. Betweenness centrality was calculated by taking the fraction of shortest paths between any two nodes that include the given node [44]. Degree and betweenness centrality outcomes were averaged by brain region: primary sensory, visual motor, motor, aPFC, FEF, and dlPFC.

### 2.7. Feature Selection

We identified several demographic, motor performance, counting performance, and fNIRS outcomes, which may be valuable features for a machine learning model to identify cognitive impairment (Table 1). Univariate analysis for potential features as independent variables was done in JMP (version 18.0.1, copyright 2024, SAS Institute Inc), and features with a significant association (*p*<0.05) with cognitive group (CNOA vs CIOA) were selected for subsequent steps. Collinearity between parameters investigated using variance inflation factor (VIF) values; a VIF cutoff value greater than 10 was considered an indication of collinearity [45]. Finally, stepwise parameter selection was used to select features based on Akaike information criterion (AIC) values.

### 2.8. Machine Learning Approach and Statistical Analysis

Three well-established machine learning algorithms were explored in this study: logistic regression (generalized linear model with binomial link), support vector machine (SVM), and an ensemble classifier using bootstrap-aggregated (bagged) decision trees. All models were developed in MATLAB (R2025B). Each model type includes its own strengths and weaknesses; logistic regression models, based on linear regression, are well explored and are easily interpretable. SVMs have been shown to be effective in processing brain signals and also has been previously explored to classify cognitive impairment using fNIRS-based features [22, 46]. Decision tree is a non-parametric supervised learning model [47]. In decision tree models, bagging can be used to reduce variance and prevent overfitting by randomly sampling from the training set and creating multiple sub datasets [48].

To evaluate the classification algorithms performance, 10-fold cross-validation was used. The full dataset (including both groups) was randomly partitioned into ten folds, with nine folds allocated for training and one-fold for testing. To ensure consistency, an equal number of participants from each group were randomly assigned to each fold. This process was repeated ten times, with each iteration utilizing a different fold for testing. There was no overlap between testing groups, such that each subject was used in the testing fold only once. Due to the randomness of data partitioning, the 10-fold cross-validation procedure was repeated 300 times. To compare classification algorithms, we computed metrics including F1 score, accuracy, and the area under the curve (AUC) of the receiver operating characteristic (ROC) curve. Metrics were averaged across the 10-folds of all 300 cross-validation iterations.

To investigate between group differences in demographic, neuropsychological test scores, and clinical questionnaire results, univariate analysis of variance (ANOVA) was used for continuous variables and chi-squared test for categorical variables.

## 3. Results

### 3.1. Participants

A total of 75 participants were included; 43 were classified as CNOA (age = 75.47 ± 7.15 years; 27 females; MoCA score = 26.56 ± 1.85), and 32 as CIOA (age = 75.90 ± 7.48 years; 22 females; MoCA score = 20.67 ± 4.87). Education level and MoCA scores were significantly different between the CNOA and CIOA groups (*p*<0.01, Table 2).

### 3.2. Machine Learning Models

The selected sensor-based features that led to the greatest model performance were the average aPFC correlation-based betweenness centrality during rest, the average aPFC coherence-based degree during rest, motor sample entropy, and the total number of correct subtractions (Table 3). Of the three models, the SVM model achieved the best average performance. The SVM model F1 score, accuracy, and AUC from cross-validation results were 68.91, 75.50, and 85.57, respectively (Table 4). The logistic regression model F1 score, accuracy, and AUC were 68.88, 75.05, and 85.50, respectively (Table 4). Lastly, the bagged decision tree model had the weakest performance, with the F1 score, accuracy, and AUC being 64.18, 69.59, and 78.02, respectively (Table 4).

SVM model performance could be further improved using education level as a demographic predictor; the SVM model F1 score, accuracy, and AUC became 70.48, 77.50, and 84.88, respectively.

## 4. Discussion

### 4.1. UEF Performance Predictors

Our study demonstrated that incorporating motor performance outcomes from the UEF dual task are useful for differentiating between CIOA and CNOA. Specifically, the key motor performance predictor was entropy of the angular velocity signal. Entropy is a measure of the uncertainty of the angular velocity and represents the elbow flexion predictability [24]. Angular velocity entropy increases with cognitive impairment, meaning that variability of motor performance is increased. Previous work has shown that decreased executive functioning is associated with increased gait variability as measured by entropy in participants with dementia [49]. Additionally, gait variability has been used to predict cerebral small vessel disease, a precursor to vascular cognitive impairment and dementia, in older adults [50]. Within our previous work, entropy was shown to be a promising predictor compared to other motor performance outcomes for use in logistic models to identify cognitive impairment in older adults [24]. Further, within our previous work, including motor entropy in addition to flexion number, flexibility variability, and flexion variability as input parameters, increased model sensitivity by 5% [24]. Including nonlinear measures may be more beneficial than standard, simple measures of motor variability, such as the coefficient of variation. Compared to these peak dependent measures, entropy considers the entire signal and can reveal hidden dynamical structures [17].

Additionally, the number of correct subtractions was the most important predictor among those of counting performance. Arithmetic is selected as the cognitive component over other comparable tasks, such as naming animals, as it is associated with working memory, semantic memory and executive functioning [51]. These processes are related to planning, initiating, and regulating coordinated actions as well as learned knowledge [52–55]. Working memory and executive functioning deficits are both common features of pathological cognitive impairment [56]. Previous work has validated an association between counting tasks and cognitive decline [57, 58]. Both UEF performance predictors (angular velocity entropy and the number of correct subtractions) support the notion that measures of impaired executive functioning are critical for identifying cognitive impairment among older adults.

### 4.2. fNIRS Predictors

The current findings suggest that incorporating measures from fNIRS recordings may be useful for differentiating between CIOA and CNOA. The most critical fNIRS predictors were resting state connectivity outcomes: average aPFC correlation-based betweenness centrality and average aPFC coherence-based degree (aPFC). Degree is a measure of how many direct connections each node has to other nodes, and lesser degree indicates decreased importance of a node [43]. Similar findings of lesser degree due to cognitive decline in older adults was reported in a resting state fMRI study [59]. Similarly, betweenness centrality quantifies how often a node lies along the shortest path between other nodes, and greater betweenness centrality conveys increased information transfer [60]. Findings of increased betweenness centrality in CIOA have been identified in resting-state EEG and magnetoencephalography (MEG) studies, such that betweenness centrality increases from CNOA to mild cognitive impairment (MCI), and from MCI to AD [61–63]. Collectively, it is suggested that early cognitive impairment is associated with distinct changes in functional connectivity profiles compared to healthy older adults [64]. These findings are supported by associations between changes in functional connectivity patterns and changes in cognitive/behavioral measures [64]. Overall, resting-state functional connectivity measures may provide a sensitive method of screening cognitive impairment.

Interestingly, the selected fNIRS predictors were all measured from channels located over the aPFC. The aPFC is known for its role in multiple functions including problem solving, memory retrieval, prospective memory, and attention allocation [65]. Additionally, the aPFC is often activated during multitasking [65]. Some of these functions are known to be impaired by dementia. Deficits in prospective memory, for example, have been reported in amnestic MCI and MCI-AD [66]. Likewise, it has also been suggested that memory retrieval deficits may occur earlier in AD than other types of memory deficits [67]. The findings of this study suggest that functional changes in this region provide valuable information to identify early forms of cognitive impairment in older adults. Focusing measurements on this region in future work can further improve the clinical viability of the proposed methods by reducing the number of required fNIRS channels as well as reducing time for experimental setup.

### 4.3. Cognitive Status Classification Using Machine Learning

In this study, we proposed a novel method for early identification of cognitive impairment in older adults using wearable sensors (fNIRS and gyroscope) during a resting state and a UEF dual task. Among the explored machine learning algorithms, SVM had the best performance with an accuracy of 77.50%. Although very little research has been done to investigate fNIRS outcomes as predictors in multiple types of supervised machine learning models to identify cognitive impairment, other disease-screening applications have demonstrated similar findings. For instance, studies that used fNIRS outcomes to differentiate between healthy individuals and those with Parkinson’s disease found superior classification using SVM [22, 68]. One notable advantage of SVMs compared to other supervised techniques is its generalizability, which makes it ideal for clinical research, especially in the case of limited sample size [69].

### 4.4. Clinical Implications

There are a few valuable clinical implications of the presented methods. Firstly, gait is the most commonly reported motor component of dual tasks for use in screening pathological cognitive impairment [16]. However, gait is suboptimal in that is common for older adults to have mobility limitations and clinics are often confined in physical space. The UEF dual task can be performed while sitting and has no fall risk. Additionally, the duration of the data collection is relatively short: 6 minutes in total, 3 minutes for a resting period and 3 minutes for the task period. In the current work, motor measures were calculated using only 60 seconds of the signal, and in future work it may be possible to shorten the duration even more, such that the task recording is only one minute long. There are a great variety of available neuropsychological examinations recommended for use in screening, leading to inconsistency and subjectivity [70]. The proposed method could be used to improve objectivity of screening. Finally, it is thought that functional changes occur far earlier in disease progression than behavioral manifestations. The proposed methods may be useful for earlier detection of cognitive impairment, allowing for a window of effective clinical intervention prior to significant disease pathology [71].

### 4.5. Limitations

A major limitation of this study is the sample size of the CNOA and CIOA groups. Small sample size is common in high dimensional datasets due to the cost of data acquisition, although it can lead to biased findings [72]. Further investigations of the proposed methods using larger sample sizes can help to improve the generalizability of the findings. Data augmentation along with deep learning approaches may also be explored in future works to help improve sample size.

Each type of dementia has unique pathology which can vary throughout disease progression. Stages and forms of dementia are not distinguished among the current participants. Future work can improve on this by using more extensive neurocognitive assessments and biomarker testing to differentiate between types of dementia [8, 73–75]. Additionally, longitudinal measurements can be taken at different stages of disease progression. In future works, fNIRS- and motion-based classification algorithms could be investigated to differentiate stages and forms of dementia.

## 5. Conclusions

The current study supports the growing literature on the use of portable functional neuroimaging techniques for use in screening cognitive impairment. A novel approach was explored, involving the use of a motion sensor-based upper extremity function dual task and resting state fNIRS measures. The selected features included motion variability and fNIRS anterior prefrontal cortex connectivity outcomes. Results of 10-fold cross validation showed the support vector machine model had the best performance compared to logistic regression and bagged decision trees with an accuracy of 75.50% and F1 score of 68.91. When used with machine learning algorithms, the UEF dual task may provide an objective method for early identification of dementia in older adults.

## Figures and Tables

**Figure 2.**
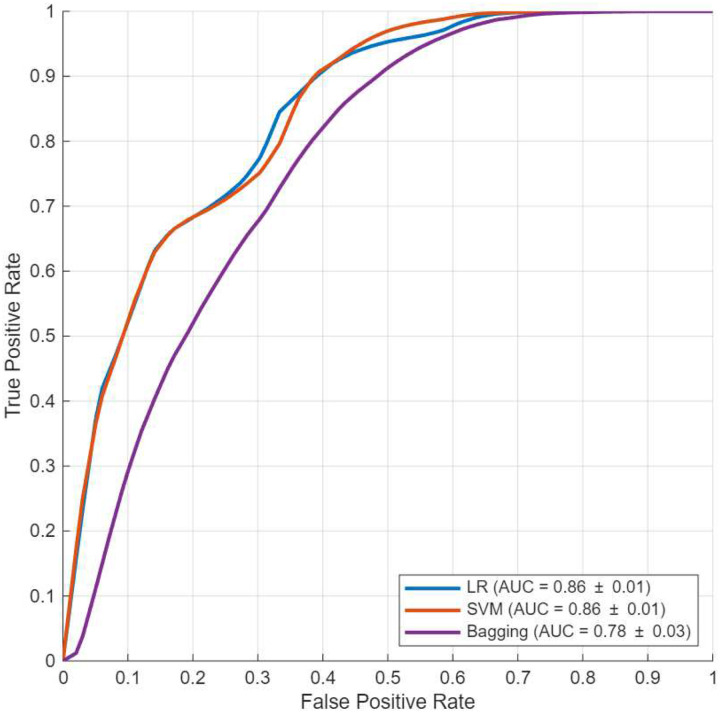
Mean receiver operator characteristic (ROC) curves for each model Abbreviations: AUC, area under the curve; LR, logistic regression; SVM, support vector machine

**Table 1.** Parameters explored using feature selection

Features	Definition
**Demographic features**	
**Age**	-
**Body mass index**	-
**Sex**	-
**Education level**	Binary measure of highest education level. 0 corresponds to high school or below, 1 corresponds to post high school and above
**Motor performance features**	
**Flexion number**	Total number of elbow flexions
**Range of motion variability**	Coefficient of variation of the flexion angle range of motion
**Flexion variability**	Coefficient of variation of the time distances between consecutive angular velocity peaks
**Motor sample entropy**	Complexity of motor data based on angular velocity
**Counting performance features**	
**Number of correct subtractions**	Number of subtracted numbers without mistakes
**Number of incorrect subtractions**	Number of subtracted numbers with mistakes
**Functional near-infrared spectroscopy features**	
**Correlation-based degree**	Number of direct between-channel functional connections (time-domain)
**Coherence-based degree**	Number of direct between-channel functional connections (frequency-domain)
**Correlation-based betweenness centrality**	Frequency that a given channel appears on the shortest path between channel pairs (time-domain)
**Coherence-based betweenness centrality**	Frequency that a given channel appears on the shortest path between channel pairs (frequency-domain)

**Table 2.** Demographic information, neuropsychological tests, and clinical measures

Demographic information	CNOA (n = 43)	CIOA (n = 32)	CNOA-CIOA *p*-value
**Age (SD)**	75.465 (7.15)	75.903 (7.48)	0.796
**BMI, kg/m^2 (SD)**	27.366 (6.36)	29.018 (5.12)	0.238
**Female, n (% of group)**	27 (62.79)	22 (68.75)	0.464
**Highest level of education, n 0 = High school or below, 1 = Post high school education**	0: 6 1: 36	0: 13 1: 19	**0.013** *
**Hispanic, n (% of group)**	3 (6.98)	5 (15.63)	0.247
**Race, n (1 = Caucasian, 2 = Asian, 3 = Black or African American)**	1: 39 2: 3 3: 0	1: 26 2: 1 3: 4	0.995
**Neuropsychological tests and clinical measures**			
**MoCA (SD)**	26.558 (1.85)	20.667 (4.87)	**<0.001** *
**CCI (SD)**	5.000 (2.64)	5.233 (2.54)	0.704
**PHQ-9 (SD)**	2.186 (3.49)	2.290 (3.37)	0.896

*Note:* A significant association of *p*<0.05 is indicated by bolded font and an asterisk (*).

Abbreviations: SD, standard deviation; CNOA, cognitively normal older adults; CIOA, cognitively impaired older adults; BMI, body mass index; MoCA, Montreal Cognitive Assessment; CCI, Charlson Comorbidity Index; PHQ-9, Patient Health Questionnaire

**Table 3.** Selected features for use in machine learning models

Features	Mean feature (SD)		CNOA-CIOA *p*-value
	CNOA (n=43)	CIOA (n=32)	
**Motor performance features**			
**Motor sample entropy**	4.473 (0.40)	4.848 (0.49)	**0.002** *
**Counting performance features**			
**Correct number of subtractions**	38.783 (20.23)	20.695 (16.55)	**<0.001** *
**Functional near-infrared spectroscopy features (fNIRS)**			
**aPFC average correlation-based betweenness centrality during rest**	1.807 (2.85)	6.004 (9.10)	**<0.001** *
**aPFC average coherence-based degree during rest**	5.268 (5.55)	2.516 (2.55)	**<0.001** *

Note: A significant association of *p*<0.05 is indicated by bolded font and an asterisk (*).

Abbreviations: CNOA, cognitively normal older adult; CIOA, cognitively impaired older adult; SD, standard deviation; aPFC, anterior prefrontal cortex

**Table 4.** Performance metrics of the machine learning models

Outcomes	Logistic Regression	SVM	Bagged Decision Tree
**F1 Score**	68.88	68.91	64.18
**Accuracy (%)**	75.05	75.50	69.59
**AUC**	85.50	85.57	78.02

Abbreviations: AUC, area under the curve

## Data Availability

The datasets generated during and analyzed during the current study are available from the corresponding author on reasonable request.
